# Supplementary material for: The vaginal microbiota, symptoms, and local immune correlates in transmasculine individuals using sustained testosterone therapy
Source: bioRxiv. 2025 Mar 15:2025.03.14.643255. Preprint. [Version 1] doi: 10.1101/2025.03.14.643255 (PMC11952554; doi:10.1101/2025.03.14.643255)
Supplement: 1 [file NIHPP2025.03.14.643255V1-supplement-1.pdf]

bioRxiv preprint doi: https://doi.org/10.1101/2025.03.14.643255; this version posted March 15, 2025. The copyright holder for this preprint (which was not certified by peer review) is the author/funder, who has granted bioRxiv a license to display the preprint in perpetuity. It is made available under aCC-BY 4.0 International license.

SUPPLEMENTARY TABLES

Table S1. Local and systemic exposures included in the analyses

Systemic Exposures	Yes	No
Current Anti-androgenic Medication Use	3 (3.5)	82 (96.5)
Probiotic Use, past 30 days	6 (7.1)	79 (92.9)
Antibiotic Use, past 30 days	3 (3.5)	82 (96.5)
Nicotine Use, past 30 days	14 (16.5)	71 (83.5)
Marijuana Use, past 30 days	36 (42.4)	49 (57.6)
**Genital Exposures**		
Current Local Estradiol Use	4 (4.7)	81 (95.3)
HPV diagnosis ever	5 (5.9)	80 (94.1)
Herpes diagnosis ever	8 (9.4)	77 (90.6)
"Bottom" Gender Affirming Surgeries	18 (21.2)	67 (78.8)
Hysterectomy and Oophorectomy	12	

bioRxiv preprint doi: https://doi.org/10.1101/2025.03.14.643255; this version posted March 15, 2025. The copyright holder for this preprint (which was not certified by peer review) is the author/funder, who has granted bioRxiv a license to display the preprint in perpetuity. It is made available under aCC-BY 4.0 International license.

Hysterectomy alone	4	
Oophorectomy alone	1	
Metoidioplasty	1	
Steroid Use, past 30 days	13 (15.3)	72 (84.7)
Current IUD	10 (11.8)	75 (88.2)
Copper IUD	3	
Progestin IUD	7	

Table S2. Self-reported symptoms experienced by transmasculine individuals currently or in the past 7 days

Self-Reported Symptoms, Past 7 days	N (%)
Dryness	20 (23.5)
Pain during Sex	11 (12.9)
Non-menstrual bleeding	5 (5.8)
Discharge	5 (5.8)
Cramping	4 (4.7)
Itching or Burning Sensation	3 (3.5)
Pain	3 (3.5)
Tissue Thinning or Tearing	3 (3.5)
"Not Normal" or Unpleasant Odor	2 (2.4)
No symptoms	48 (56.5)

Table S3. Immune analytes characterized and detected in transmasculine individuals and reproductive-age cis women

The Kruskal-Wallis test was used to test for differences in distribution.

	Transmasculine Individuals		Reproductive-Aged Cis Women		KW p-value
	Number of Samples Detected In, n (%)	TM, median (range)	Number of Samples Detected In, n (%)	CF, median (range)	
IL-8	208 (100)	2590.6 (3.5 - 6442.5)	29 (100)	2864.7 (132.3 - 7799.9)	0.3228
IL-1α	207 (99.5)	723.9 (6.8 - 17195.5)	29 (100)	1753.0 (431.2 - 19901.9)	**0.000308**
MIG	205 (98.6)	656.1 (7.5 - 10993.0)	29 (100)	704.2 (22.2 - 8375.3)	0.2081
IL-6	204 (98.1)	25.6 (1.7 - 1494.3)	29 (100)	24.8 (2.2 - 1583.9)	0.6802
RANTES	203 (97.6)	95.1 (2.4 - 1293.6)	29 (100)	9.5 (3.0 - 428.9)	**9.659E-09**
IL-1β	203 (97.6)	130.1 (5.8 - 13264.7)	29 (100)	73.7 (10.2 - 1211.9)	0.08018

bioRxiv preprint doi: https://doi.org/10.1101/2025.03.14.643255; this version posted March 15, 2025. The copyright holder for this preprint (which was not certified by peer review) is the author/funder, who has granted bioRxiv a license to display the preprint in perpetuity. It is made available under aCC-BY 4.0 International license.

MIP-1β	201 (96.6)	54.7 (7.2 - 1345.8)	29 (100)	5.3 (5.3 - 103.0)	**1.682E-10**
IL-10	195 (93.8)	44.6 (3.6 - 1198.1)	19 (65.5)	4.9 (3.6 - 45.6)	**9.772E-09**
TNF-α	177 (85.1)	26.7 (7.5 - 574.5)	12 (41.4)	9.2 (7.6 - 45.1)	**0.0000232**
IP-10	172 (82.7)	56.0 (7.0 - 3723.9)	28 (96.6)	158.9 (3.8 - 1249.7)	**0.000258**
IFN-γ	122 (58.7)	14.8 (7.5 - 66.5)	21 (72.4)	12.7 (7.5 - 26.9)	0.254
MIP-1α	117 (56.2)	157.4 (18.2 - 1856.0)	9 (31)	39.7 (22.8 - 229.4)	**0.0061**
IL-22	68 (32.7)	95.3 (65.5 - 187.3)	10 (34.5)	107.1 (69.1 - 167.3)	0.1835

Table S4. Stability of transmasculine individual vaginal microbiome over one week, as measured by the Yue-Clayton theta index

Rows are the starting tmCST and columns are the ending tmCST for paired samples from the same individual. Yue-Clayton theta indices for each tmCST transition were averaged and the range displayed.

	1	2	3	4	5	6	7	8	9	10	11
1	0.786 (0.423-0.997)	-	-	-	-	-	-	-	-	-	-
2	-	0.615 (0.325-0.822)	-	0.251 (0.251-0.251)	-	-	-	-	0.113 (0.113-0.113)	-	-
3	0.098 (0.098-0.098)	-	0.617 (0.259-0.85)	0.212 (0.212-0.212)	-	-	-	-	0 (0-0)	-	-
4	-	0.332 (0.227-0.465)	0.546 (0.546-0.546)	0.488 (0.068-0.929)	0.262 (0.262-0.262)	-	-	0.409 (0.409-0.409)	0.027 (0.027-0.027)	0.305 (0.305-0.305)	-
5	-	-	-	0.132 (0.132-0.132)	0.453 (0.128-0.626)	-	0.396 (0.076-0.678)	0.129 (0.129-0.129)	0.103 (0.103-0.103)	-	-
6	-	-	-	-	0.506 (0.506-0.506)	0.583 (0.427-0.725)	0.093 (0.093-0.093)	-	-	-	-
7	0.528 (0.528-0.528)	0.005 (0.005-0.005)	-	-	0.225 (0.075-0.522)	-	0.437 (0.039-0.856)	0.556 (0.556-0.556)	0.26 (0.038-0.559)	0.438 (0.311-0.565)	-
8	-	-	-	-	-	-	0.055 (0.02-0.089)	0.536 (0.24-0.743)	0.563 (0.563-0.563)	0.189 (0.189-0.189)	-
9	-	-	0.1 (0.1-0.1)	-	0.041 (0.041-0.041)	-	0.379 (0.379-0.379)	-	0.518 (0.246-0.732)	0.691 (0.691-0.691)	-
10	-	-	-	0.134 (0.039-0.37)	-	-	0.212 (0.045-0.341)	0.489 (0.394-0.584)	0.222 (0.222-0.222)	0.661 (0.424-0.962)	-
11	-	-	-	-	-	-	-	-	-	-	0.138 (0.044-

bioRxiv preprint doi: https://doi.org/10.1101/2025.03.14.643255; this version posted March 15, 2025. The copyright holder for this preprint (which was not certified by peer review) is the author/funder, who has granted bioRxiv a license to display the preprint in perpetuity. It is made available under aCC-BY 4.0 International license.

											0.232)
